# Genetic Diversity and Differentiation of the Orange-Spotted Grouper (*Epinephelus coioides*) Between and Within Cultured Stocks and Wild Populations Inferred from Microsatellite DNA Analysis

**DOI:** 10.3390/ijms12074378

**Published:** 2011-07-06

**Authors:** Le Wang, Zining Meng, Xiaochun Liu, Yong Zhang, Haoran Lin

**Affiliations:** State Key Laboratory of Biocontrol, Institute of Aquatic Economic Animals and the Guangdong Province Key Laboratory for Aquatic Economic Animals, School of Life Sciences, Sun Yat-Sen University, Guangzhou 510275, China; E-Mails: wangle@student.sysu.edu.cn (L.W.); lsslxc@mail.sysu.edu.cn (X.L.); lsszy@mail.sysu.edu.cn (Y.Z.); lsslhr@mail.sysu.edu.cn (H.L.)

**Keywords:** the orange-spotted grouper, genetic diversity, differentiation, microsatellite, *Epinephelus coioides*

## Abstract

In the present study, we employed microsatellite DNA markers to analyze the genetic diversity and differentiation between and within cultured stocks and wild populations of the orange-spotted grouper originating from the South China Sea and Southeast Asia. Compared to wild populations, genetic changes including reduced genetic diversity and significant differentiation have taken place in cultured grouper stocks, as shown by allele richness and heterozygosity studies, pairwise F_st_, structure, molecular variance analysis, as well as multidimensional scaling analysis. Although two geographically adjacent orange-spotted grouper populations in China showed negligible genetic divergence, significant population differentiation was observed in wild grouper populations distributed in a wide geographical area from China, through Malaysia to Indonesia. However, the Mantel test rejected the isolation-by-distance model of genetic structure, which indicated the genetic differentiation among the populations could result from the co-effects of various factors, such as historical dispersal, local environment, ocean currents, river flows and island blocks. Our results demonstrated that microsatellite markers could be suitable not only for genetic monitoring cultured stocks but also for revealing the population structuring of wild orange-spotted grouper populations. Meanwhile, our study provided important information for breeding programs, management of cultured stocks and conservation of wild populations of the orange-spotted grouper.

## 1. Introduction

The orange-spotted grouper (*Epinephelus coioides*), an economically high valued marine food fish species belongs to the subfamily Epinephelinae (family, Serranidae) [1], which inhabits in a large area from eastern Africa, south to at least Durban (South Africa), east to the western Pacific from the Ryukyu Islands to Australia, and eastward to Palau and Fiji [2]. However, due to overfishing and habitat destruction, the wild populations of the orange-spotted grouper have declined greatly in recent years and this species has been classified as nearly threatened [3]. Over the last decades, various attempts have been made to conserve this grouper species. The most important achievement is successful practices in aquaculture for the orange-spotted grouper, which can alleviate the fishing pressure on wild populations [4]. According to the FAO fishery statistics, the global aquaculture production of the orange-spotted grouper has dramatically increased nearly 40-fold between 1999 and 2008 [5]. At present, it has become one of the grouper species most commonly cultured on commercial scales in Asian-Pacific region and a major food fish in Hong Kong live fish markets in China [6].

However, aquaculture practices are likely to reduce the genetic diversity by founder effects, inbreeding and random genetic drift in hatchery-reared stocks, which may cause loss of disease resistance and reduce environmental adaptability, thus limit the genetic potential for selective breeding [7–9]. For this reason, some research reports focused on genetic monitoring of the differences between hatchery stocks and wild populations in various fish species including Turbot (*Scophthalmus maximus*), Atlantic salmon (*Salmo salar*), Sea bream (*Pagrus major*), Japanese flounder (*Paralichthys olivaceus*), Arctic charr (*Salvelinus alpinus*), common carp (*Cyprinus carpio*) using DNA fingerprinting techniques such as microsatellite and mitochondrial markers [10–15]. Unfortunately, despite the importance of orange-spotted grouper for commercial aquaculture in Southeast Asia, literature about its genetic background is scarce and mainly focuses on the genetic differentiation among geographical populations [16,17], therefore the genetic difference between cultured stocks and wild populations is still unknown.

Marine fishes are considered to be genetically less differentiated compared to freshwater fishes due to high gene flow in open marine environment [18]. The dispersal potential of planktonic eggs and larvae and the absence of geographical barriers between different populations all contribute low genetic differentiation in marine fishes. Nevertheless, the study on population genetic structure of marine fishes is crucial and can provide important information for conservation and management of the genetically different populations. Meanwhile, such study can also suggest essential theoretical and practical guidance for improved breeding programs, as the genetically differentiated populations have the potential to be used for hybridization to improve the genetic quality of cultured fish [19,20].

We have recently isolated polymorphic microsatellite markers and shown that these markers have the potential to characterize the genetic diversity and analyze the population structure of the orange-spotted grouper [21]. In the present study, we employed microsatellite DNA markers to compare the genetic diversity and differentiation between and within cultured stocks and wild populations of the orange-spotted grouper originating from the South China Sea and Southeast Asia including Malaysia and Indonesia. Our goals were to evaluate the putative genetic changes in cultured stocks as a result of founder effect, random genetic drift and inbreeding during aquaculture practices compared to wild populations, and examine the population differentiation within samples distributed in a wide area from China, through Malaysia to Indonesia in order to facilitate the breeding programs of the orange-spotted grouper.

## 2. Materials and Methods

### 2.1. Sample Collection and DNA Extraction

**Figure 1:**
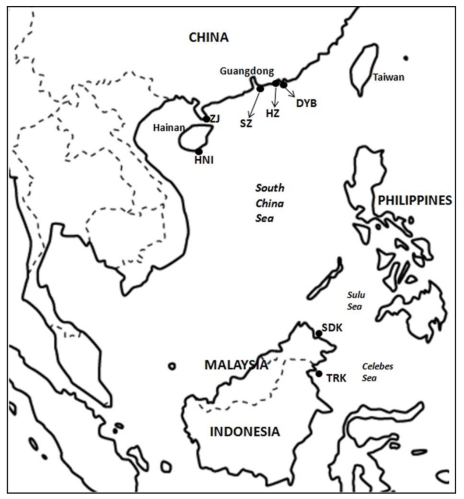
Sampling locations of the orange-spotted grouper in China and the Southeast Asia. HZ: cultured stock in Daya Bay, Huizhou; SZ: cultured stock in Shenzhen; ZJ: cultured stock in Zhanjiang; DYB: wild population in Daya Bay; HNI: wild population in Hainan Island; SDK: wild population in Sandakan, Malaysia; TRK: wild population in Tarakan, Indonesia.

Our samples were mainly obtained from the South China Sea, from which almost all the cultured and wild orange-spotted grouper in China mainland presently originated, due to its ideal subtropical climate. Sampling locations are shown in Figure 1. In this study, all samples from China, including three cultured stocks (HZ, SZ and ZJ) and two wild populations (DYB and HNI) were acquired within this region. In detail, HZ stock (n = 26) was the first generation progeny of a broodstock reared in a government-sponsored hatchery (Guangdong Daya Bay Fisheries Development Center, Huizhou City, China). Both SZ (n = 15) and ZJ (n = 34) stocks were also the first generation offspring from private hatcheries located in Shenzhen and Zhanjiang Cities, respectively. These hatcheries were small- or medium-sized with a limited number of orange-spotted grouper. Both wild caught and captive cultured sources of the broodstock were investigated. In detail, large-sized groupers in the wild were collected during the spawning season and treated as broodstock directly. Alternatively, captured juveniles were cultured in captivity until sexual maturation and then used as broodstock. However, information about the exact proportion of these two different sources of individuals in each broodstock and management practices on cultured stocks were not available. Two wild populations of China, DYB (n = 50) and HNI (n = 40), were caught in the South China Sea near Daya Bay and Hainan Island, respectively. Furthermore, another two wild populations collected in the Southeast Asia were used as ‘outgroup’ for comparison with the Chinese samples, including the Sandakan population of Malaysia (SDK, n = 25) and Tarakan population of Indonesia (TRK, n = 11). All samples of the wild orange-spotted grouper were caught by hook and line in 2009.

Caudal fin or muscle tissue samples of the orange-spotted grouper were clipped and immediately preserved in 90% ethanol until DNA extraction was performed. Genomic DNA was isolated according to the phenol-chloroform extraction protocol by Hoelzel and Green [22].

### 2.2. Microsatellite Genotyping

A total of 11 microsatellite loci were selected for genotyping fish individuals from the seven samples (Table 1). Forward primers were 5′-labeled with a fluorescent dye HEX or 6-FAM. PCR amplification was performed on PTC-200 thermal cycler (MJ Research) in a 20-μL volume containing 0.5 μm of each primer, 0.2 mM dNTP, 1.5 mM MgCl_2_, 1XPCR buffer, 1U Taq DNA polymerase (Fermentas), and 20ng template genomic DNA. PCR program was as follows: 5 min at 94 °C followed by 30 cycles of 30 s at 94 °C, 30 s at the annealing temperature (Table 1), and 30 s at 72 °C with a final extension of 5 min at 72 °C. PCR products were separated on an ABI PRISM 3730 DNA automated sequencer (Applied Biosystems). Fragment size was measured according to the ROX-500 standard using GeneMapper (Applied Biosystems). Genotype data was exported to Excel tables for analysis. MicroChecker [28] was used to check the genotyping errors and possible presence of null alleles.

### 2.3. Statistical Analysis

Intra-population genetic diversity was assessed by computing the number of alleles per locus (A), Allelic richness (Ar), observed heterozygosity (Ho) and expected heterozygosity (He) using the program FSTAT 2.9.3 [29]. Since allele number is influenced by size of different samples, we used allele richness for comparison in this study [30]. Differences in genetic diversity parameters between cultured stocks and wild populations were tested using nonparametric analysis (Mann-Whitney U test; [31]). The software GENEPOP 4.0 computer package [32] was used to test linkage disequilibrium between pairwise loci and departure from Hardy-weinberg equilibrium (HWE) across all loci. The Markov chain method was employed to calculate an unbiased estimate of the p-value in order to test deficiency or excess of heterozygote with the following parameters (dememorization = 1000, batches = 500, and iterations per batches = 1000). Sequential Bonferroni correction was used to adjust the significance of HWE and linkage disequilibrium tests [33]. The program BOTTLENECK version 1.2.02 [34] was used under the infinite allele model (IAM), stepwise-mutation model (SMM) and two-phase model (TPM) with 1000 iterations to check the existence of bottleneck inferred by heterozygosity excess in the seven samples. The significance was tested using Wilcoxon sign rank test. Following the recommendations of Kuikart & Cornuet [34], if significant results were obtained under all these three models, populations can be concluded as having experienced bottleneck. Inbreeding coefficients (ƒ) were also calculated using the program GDA [35].

Inter-population genetic differentiation was estimated with pairwise Fst values and significance tests of pairwise Fst was computed by a permutation with 10,000 replicates using ARLEQUIN 3.11 [36]. Furthermore, the genetic relationship among samples was visualized by MDS (Multidimensional scaling) based on pairwise Fst values using SPSS 13.0. Analysis of molecular variance (AMOVA) was also calculated using ARLEQUIN 3.11 [36]. AMOVA was used to partition genetic variance hierarchically between the wild group and cultured group. However, AMOVA was not used to partition wild groups in different geographical regions (China, Malaysia and Indonesia). As two of these three groups consisted of a single population, this would influence the analysis in a discriminatory way. The correlation between genetic distance and geographical distance was evaluated using the Mantel test by the program TFPGA version 1.3 [37]. We also analyzed the population structure of all samples using the program Structure 2.2.3 [38]. This program was used to infer the number of putative clusters (K) and assign individuals into corresponding clusters. We performed this analysis under admixture model and using 10^5^ iterations after a 10^5^ burn-in length with K ranging from 1 to 7. The most likely K value was inferred by calculating ΔK using the method of Evanno *et al.* [39].

## 3. Results

### 3.1. Polymorphisms of Microsatellites

Microchecker analysis showed no evidence for null alleles and allele stuttering. Examination of the Hardy-Weinberg equilibrium (HWE) showed that two loci (CA-6 and Ec_157) [26,21] exhibited non conformity to HWE in many samples. Therefore, these two loci were excluded for subsequent analysis. Among 63 locus population cases, 11 cases significantly deviated from HWE. After sequential Bonferroni correction, only three cases showed significant (Table 2). Examination of linkage disequilibrium using Fisher’s exact test showed no disequilibrium in these microsatellite loci. A total of 165 alleles were detected across nine microsatellite loci, all of which were polymorphic. The number of alleles per locus ranged from 4.286 (GAA-1) to 17.143 (D496) with an average value of 9.651. The expected heterozygosity (H_e_) at each locus varied from 0.460 (Mbo061) to 0.916 (D496) with a mean value of 0.716.

### 3.2. Intra-Population Genetic Diversity

For the Chinese samples, a statistically significant (P < 0.05) reduction of allele richness (Ar) was observed in cultured stocks of orange-spotted grouper (Ar between 5.176 and 6.674, mean = 5.821) compared to the wild populations (Ar between 7.176 and 7.454, mean = 7.311) (Table 2). The mean expected heterozygosity (He) in the two groups (Cultured and Wild) were 0.689 and 0.748, respectively. However, genetic diversity in terms of heterozygosity was not markedly reduced (P > 0.05) in contrast to the significant reduction of Ar. The Inbreeding coefficient (ƒ) value in the cultured stocks did not show significant differences with that of the wild populations of the Chinese samples (P > 0.05) (Table 2). On the other hand, for the wild populations originating from different geographical areas, the highest level of genetic diversity was detected in Malaysian population SDK (Ar = 8.421, He = 0.812), and the lowest was in Indonesian population TRK (Ar = 6.367, He = 0.641) (Table 2). Bottleneck analysis showed no evidence of recent bottleneck for any of the seven samples except for the HZ stock, which showed heterozygosity excess under IAM of microsatellites (P = 0.037). The results of bottleneck analysis most likely indicate that all these seven samples have not experienced recent bottleneck.

### 3.3. Inter-Population Genetic Differentiation

**Figure 2:**
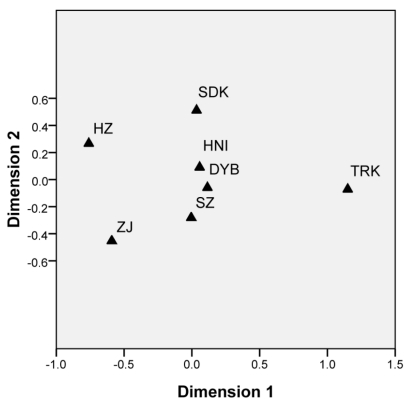
Multi-dimensional scaling plot of four wild populations and three cultured stocks of the orange-spotted grouper for genetic distribution based on pairwise Fst values.

**Figure 3:**
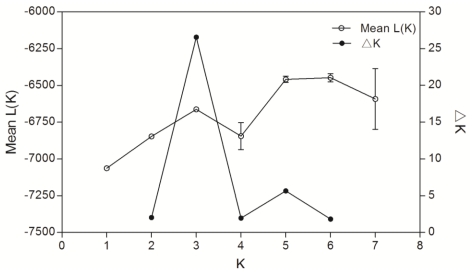
The log probability of data [L(K) ± SD] over 20 runs for each K and ΔK values, where the highest level of structure is suggested to be the true number of clusters.

**Figure 4:**
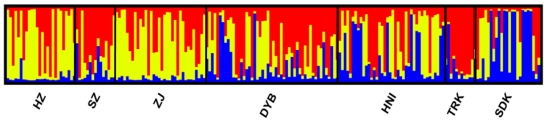
Bayesian analysis of the genetic structre based on nine microsatellite loci. Each individual is represented by a vertical line, which is coloured according to the assigned groups at estimated K = 3 (see Figure 3).

Pairwise Fst analysis showed significant genetic structure among all the seven samples except two comparisons where the differentiation was not statistically significant (Table 3). The genetic differentiation between the cultured stocks and the wild populations was statistically significant (Fst = 0.024, P < 0.05), although the differentiation between cultured stock SZ and wild population DYB was not significant (P > 0.05, Table 3). For the two ‘outgroup’ samples, Malaysia SDK and Indonesia TRK not only differentiated with each other (Fst = 0.075, P < 0.05), but also significantly differentiated (0.020 < Fst < 0.049, P < 0.05) with the other samples. The largest differentiation was between the wild population TRK and the cultured stock HZ (Fst = 0.129, P < 0.05), whereas the smallest divergence was presented between the two Chinese wild populations DYB and HNI (Fst = 0.002, P > 0.05). The genetic relationships suggested by pairwise Fst values were clearly visualized in the multidimensional scaling analysis. MDS results (Stress = 0.048; RSQ = 0.985) showed the cultured stocks except for SZ markedly differentiated from the wild populations (Figure 2) and also supported the ‘outgroup’ samples (SDK and TRK) differentiated from the other samples. In simulations of the Bayesian clustering method with the program Structure, the mean L(K) and ΔK all suggested three clusters as the most likely population structure, as discontinuity was presented at this K/ΔK value (Figure 3). In the results, the cultured stocks except for SZ and wild populations showed much different plotting, and SDK and TRK also showed markedly different plotting to the other samples and to one another (Figure 4). This pattern of structuring was similar to that of MDS analysis. The Mantel test showed no significant relationship between genetic distance and geographical distance among the four wild populations (r = −0.342, Z = 224.016, upper tail P = 0.779 and lower tail P = 0.221), rejecting the isolation-by-distance model of genetic structure. AMOVA results showed that statistically significant variation occurred among populations within groups (2.987 %, P < 0.05) and between cultured and wild groups of the orange-spotted grouper in China (1.381 %, P < 0.05; Table 4). This result was consistent with that of Fst analysis.

## 4. Discussion

### 4.1. Microsatellites Polymorphism

This is the first study of genetic diversity and differentiation of the orange-spotted grouper between cultured stocks and wild populations using microsatellite markers. In this study, we found that the number of alleles per locus varied between 4.286 and 17.143, and the expected heterozygosity varied from 0.460 to 0.916 (Table 2). The polymorphism level of these microsatellite loci in the orange-spotted grouper was similar to that of some other marine fish species, such as cod (*Gadus morhua*) [40], sea bream [41], king fish (*Seriola lalandi*) [42] and Asian seabass (*Lates calcarifer*) [19], suggesting that these polymorphic microsatellites were sufficient to reveal intraspecific diversity of the orange-spotted grouper. Also, the polymorphism level detected in this study is much higher than that in previous studies that employ microsatellite analysis of the orange-spotted grouper, for two reasons [16,43]. Firstly, we employed more microsatellite loci and each of these loci had a high polymorphism level. Secondly, we used an automatic DNA sequencer to isolate PCR products and genotype, thus could find more alleles than the conventional silver-staining method [44].

### 4.2. Genetic Variability Between Cultured Stocks and Wild Populations

Several studies have shown that aquaculture practices reduce genetic variability in hatchery-reared stocks of various fish species [13–15]. As high genetic variation was considered to be related to the adaptive fitness in changing environments, losing genetic variation was detrimental to the domestication process of cultured stocks [45]. In the present study, significantly reduced genetic diversity in terms of Ar was also observed in cultured stocks of the orange-spotted grouper originating from the South China Sea, when compared to the wild populations of the same origin (Table 2). However, the reduction of He in cultured stocks was not statistically significant, though it seemed to have declined (Table 2). This non significant reduction in heterozygosity in cultured stocks in contrast to wild populations was also reported in other studies [10,11,46]. Allele richness is a more sensitive measure of genetic perturbations than heterozygosity [7,47]. Loss of rare alleles in populations can greatly influence Ar. However, this loss has little effect on heterozygosity. This has been backed up by previous theoretical and empirical studies [11,48,49]. In our study, the decline of genetic variation in cultured stocks could be caused mainly by founder effects. Founder effects could have occurred in breeding programs by using broodstock with a small number of individuals, which led to the loss of genetic diversity in cultured stocks. The founding of a stock with broodstock comprised of a few wild individuals is prone to have great effect on genetic diversity, especially on allelic diversity [50]. It is a fact that most of the hatcheries of the orange-spotted grouper in the region of southern China are small- or medium-sized with inadequate parental fish. This is mainly because the wild spawners, as the major source of broodstock in hatchery, were difficult to catch because of overfishing and habitat destruction. The decline of genetic variation in cultured stocks may be caused by the increased effect of genetic drift resulting from using a small number of parental individuals [11]. Besides founder effects, the genetic changes in cultured stocks compared to wild populations are likely due to artificial and natural selection existing in the culture environment [51,46]. However this selection could not be reflected using neutral markers as microsatellites in our study.

With respect to genetic variation changes of the cultured stocks relative to wild populations, we should not neglect the stock enhancement programs for some grouper species in China. In recent years, the government-sponsored hatcheries of the orange-spotted grouper have released a number of hatchery-reared fingerlings into the South China Sea [52]. However, the fingerlings’ genetic characteristics and adaptability to a wild environment were never assessed. If a large number of cultured groupers with reduced genetic variation compared to the wild populations were released into the South China Sea, the genetic composition of the wild populations in this area is likely to be effected. Therefore, these programs should be carried out with caution.

Genetic analysis involving allele richness and heterozygosity showed that genetic diversity among the orange-spotted grouper, dusky grouper (*Epinephelus marginatus*) and Hawaiian grouper (*Epinephelus quernus*) was comparable [53,26]. However, compared to migratory fishes, such as Atlantic cod [40], red sea bream [41] and king fish [37], these epinephelus fishes are less diverse. This might indicate that populations of the orange-spotted grouper are small and isolated [16]. The wild populations in China (HNI and DYB) had an intermediate value of genetic variability compared to the two ‘outgroup’ populations, being lower than the Malaysian SDK population but higher than Indonesian TRK population (Table 2). Based on this result, we conclude that the present wild population of the orange-spotted grouper in the South China Sea still maintain a normal level of genetic diversity and the population’s genetic variability is relatively unaffected, despite the population decline during the past decades owing to overfishing and habitat destruction. However, such conclusions must be made with caution, because the genetic diversity information of the wild grouper populations of China prior to population decline was not available. Consequently, a firm demonstration of this conclusion must be made according to further genetic analysis of historical and contemporary samples before and after population decline. Also, a reasonable sampling strategy encompassing sample size and distribution is needed in order to obtain more credible analytical results. Nevertheless, our results of the genetic variability between cultured stocks and wild populations of the orange-spotted grouper provided important information for aquaculture practices of this species, which was never studied before. Future studies on genetic diversity of this species should include more cultured samples and wild populations in other regions around the South China Sea to acquire a more comprehensive picture of the genetic variation of this species.

### 4.3. Genetic Differentiation Between and Within Cultured Stocks and Wild Populations

Pairwise Fst, Structure and AMOVA analysis revealed significant differentiation between the cultured stocks and wild populations (Table 3, Figure 4, Table 4). Significant differentiation between cultured stocks and wild populations was also observed in other fishes, such as salmon (*Salmo salar*) [11], grass carp (*Ctenopharyngodon idella*) [54,55] and Asian seabass [19]. The genetic divergence between cultured stocks and wild populations of the orange-spotted grouper may be caused by artificial selection, founder effects and random genetic drift in the cultured stocks or in the breeding program. However, artificial selection could have a minor effect on such differentiation, as the cultured stocks in our study have not been selected extensively. Founder effects should not be ruled out, because the broodstock in the hatcheries were comprised of only a few wild individuals as described above. As no evidence of bottleneck was detected in the cultured stocks, these founder effects could have been insignificant. On the other hand, the effect of random genetic drift could be an important reason for the significant differentiation. It was suggested that genetic differentiation was more influenced by a random genetic drift in a small sample than a big one [56]. The random genetic drift may also be caused by unequal sex ratio or differential reproductive contributions of the brooders in the hatcheries [57]. The orange-spotted grouper is such a species with unequal sex ratio due to late sex reversal.

Regarding the differentiation among wild populations, the insignificant differentiation between two Chinese wild populations (HNI and DYB) indicated that these two samples should be considered as one population (Table 3). This result is very consistent with a previous study of the orange-spotted grouper that no obvious difference (F_st_ = 0.011) was observed between two wild populations collected in different locations (Taiwan and Guangdong) of the South China Sea [17]. However, beyond the range of the South China Sea, Chinese wild grouper populations were significantly divergent from the two ‘outgroup’ populations, SDK (Malaysia) and TRK (Indonesia), according to F_st_ and Structure analysis (Table 3, Figure 4). Significant population structuring in a wide range of geographical areas was also observed in other grouper species, such as humpback grouper (*Cromileptes altivelis*) [58], dusky grouper [53], Rock grouper (*Epinephelus adscensionis*) [59]. This differentiation might be caused by many factors, such as biological factors including larvae dispersing ability, site fidelity, ocean currents and so on. Although the historical dispersal dynamics is a determinant key in spatially structured populations [60], it is beyond the scope of this study. The elucidation of all these factors needs a more detailed phylogeographical study.

The detection of differentiation between and within cultured stocks and wild populations in our study provided much valuable information for the breeding program of this species. The significant genetic differentiation among these wild populations might be used to produce hybrid vigour, although further experiments are required. At the same time, our results supplemented the previous population genetics studies, which showed important implications for the conservation of this species.

## 5. Conclusions

To our knowledge, this study was the first genetic monitoring research for cultured stocks and wild populations of the orange-spotted grouper. Our work demonstrated that genetic changes, including reduced genetic diversity and significant differentiation, have taken place in cultured grouper stocks compared to the wild populations due to founder effects and random genetic drifts during aquaculture practices. This result provided important information for ongoing breeding programs and stock enhancement programs. We also found considerable population differentiation among the orange-spotted grouper populations distributed in a wide geographical area from China, through Malaysia to Indonesia, although negligible genetic differentiation was observed between Chinese populations. This could result from the co-effects of various factors, but not merely a matter of isolation-by-distance. The genetic differences among geographical populations could provide more choice for the selective breeding work of the orange-spotted grouper.

## Figures and Tables

**Table 1:** Sequence of 11 pairs of microsatellite primers.

Locus	GenBank no.	Primer sequence (5′-3′)	Tann (°C)	Reference
D496	DQ914905	F: TTACTGGCAGCAATGGAC	50	[23]
		R: GATGTATGACTACGAATGG		
Mbo061	AY063512	F: TGAAGAATGTCAGATATTTTGTGGTG	53	[24]
		R: TCCCAAGAGTGTTGAAGTTATAGG		
Pm12	AY688385	F: AGAAAAAGCTCCACAACACAACAA	55	[25]
		R: GAGCCCCAGTCCCAAATATTG		
CA-2	AF539606	F: GACTTGATTCAGCAAAATAAAGATG	55	[26]
		R: AGAGACGGTGCCAGTAAATGAA		
CA-6	AF539608	F: GTGTTGCTGGGGTTACTAATGAAG	50	[26]
		R: TTAGACACATTGTCACGATGGTCC		
GAA-1	AF539612	F: GGAGTGTTAAATATGCCCACCA	60	[26]
		R: CAGAAATCGCTGAGGACAAGAG		
RH_GATA_003	DQ223790	F: GGGCAATTTGGTTCTTCACA	57	[27]
		R: TGTCAATGCCACAGGATACA		
Ec_122	GQ267997	F: CATTCCTTAAAGTATTCTGTG	55	[21]
		R: CCACAGCCAGTCTAGGTATTC		
Ec_154	GQ429007	F: AGCTGCTCAACAGGTTGTGTT	56	[21]
		R: CAAGTTCCATATGTGCTCTGACA		
Ec_157	GQ429008	F: TGGAACAAGTTGGCATGGTA	56	[21]
		R: CAAATACAACACCCTAGATTTT		
Ec_158	GQ429009	F: TGAGAGACAGTGGAGCACAAA	56	[21]
		R: CGTGGTTACATTCTACCCCCTA		

Tann, annealing temperature.

**Table 2:** Summary statistics of microsatellites in orange-spotted grouper.

Locus		HZ n = 26	SZ n = 15	ZJ n = 34	DYB n = 50	HNI n = 40	TRK n = 11	SDK n = 25	Mean
D496	A	13	12	17	23	25	13	17	17.143
	Ar	9.417	10.691	9.031	12.647	12.764	12.260	11.542	11.193
	He	0.900	0.926	0.831	0.945	0.945	0.931	0.934	0.916
	ƒ	0.063	0.062	0.053	0.052	0.045	0.047	0.038	
	P	0.185	0.917	0.270	0.418	0.450	0.091	0.061	
Mbo061	A	4	5	6	10	11	5	6	6.714
	Ar	2.692	3.992	3.974	4.930	5.508	4.900	4.247	4.320
	He	0.246	0.405	0.343	0.459	0.518	0.584	0.667	0.460
	ƒ	−0.100	−0.095	−0.097	−0.087	−0.081	−0.087	−0.148	
	P	1.000	1.000	1.000	0.897	0.948	1.000	0.000*	
Pm12	A	6	8	8	8	8	7	9	7.714
	Ar	4.634	7.285	5.469	6.528	6.513	6.810	6.674	6.273
	He	0.727	0.871	0.612	0.826	0.784	0.831	0.811	0.780
	ƒ	0.061	0.036	0.047	0.042	−0.011	0.027	0.027	
	P	0.227	0.395	0.173	0.305	0.001	0.351	0.036	
CA-2	A	4	6	5	9	10	3	18	7.857
	Ar	3.973	5.565	3.470	4.894	5.842	2.818	10.627	5.313
	He	0.732	0.735	0.485	0.607	0.672	0.177	0.886	0.613
	ƒ	0.021	0.043	0.044	0.020	0.034	0.025	−0.009	
	P	0.522	0.922	0.135	0.657	0.553	1.000	0.001	
GAA-1	A	3	6	7	3	6	2	3	4.286
	Ar	2.999	4.896	4.460	2.954	3.704	2.000	2.979	3.427
	He	0.657	0.598	0.612	0.534	0.606	0.247	0.549	0.543
	ƒ	−0.008	−0.006	0.006	0.019	0.046	0.014	0.016	
	P	0.420	0.149	0.101	0.900	0.175	1.000	0.842	
RH_GATA_003	A	7	5	8	11	9	5	7	7.429
	Ar	5.188	4.526	4.803	5.908	6.277	5.000	5.462	5.309
	He	0.698	0.540	0.648	0.672	0.731	0.616	0.743	0.664
	ƒ	−0.020	−0.018	0.017	−0.027	−0.032	−0.034	0.003	
	P	0.417	0.510	0.302	0.131	0.013	0.131	0.478	
Ec_122	A	10	11	11	15	17	11	16	13.000
	Ar	7.451	9.297	7.924	9.507	9.707	11.528	10.475	9.413
	He	0.808	0.880	0.847	0.887	0.876	0.944	0.883	0.875
	ƒ	−0.006	−0.032	−0.019	−0.050	−0.044	−0.039	−0.054	
	P	0.034	0.395	0.046	0.953	0.749	0.655	0.062	
Ec_154	A	9	7	10	20	18	7	21	13.143
	Ar	6.921	6.118	6.021	9.872	9.771	7.000	12.842	8.364
	He	0.794	0.738	0.740	0.888	0.896	0.726	0.944	0.818
	ƒ	−0.087	−0.091	−0.065	−0.169	−0.138	−0.109	−0.117	
	P	0.014	0.053	0.000*	0.094	0.792	0.976	0.873	
Ec_158	A	4	9	7	13	12	5	17	9.571
	Ar	3.307	7.695	5.367	7.265	7.003	4.991	10.944	6.653
	He	0.608	0.874	0.758	0.821	0.793	0.714	0.887	0.779
	ƒ	0.040	0.038	0.052	0.012	0.014	0.038	0.047	
	P	0.096	0.748	0.035	0.078	0.125	0.698	0.000*	
Mean	A	6.667	7.667	8.778	12.444	12.889	6.444	12.667	
	Ar	5.176	6.674	5.613	7.167	7.454	6.367	8.421	
	He	0.686	0.730	0.653	0.738	0.758	0.641	0.812	

A, number of alleles; Ar, allele richness; He, expected heterozygosity; ƒ, inbreeding coefficient; P, Hardy-Weinberg equilibrium P value;

* significant departure from HWE after sequential Bonferroni correction.

**Table 3:** Matrix of pairwise Fst values (below diagonal) and P value (above diagonal) among four wild populations and three cultured stocks of the orange-spotted grouper based on nine microsatellite loci (Correction for multiple comparison: P < 0.0024).

Pop	HZ	SZ	ZJ	DYB	HNI	TRK	SDK
HZ	0	0.000	0.000	0.000	0.000	0.000	0.000
SZ	0.053	0	0.000	0.063	0.000	0.000	0.000
ZJ	0.044	0.039	0	0.000	0.000	0.000	0.000
DYB	0.043	0.010	0.032	0	0.279	0.000	0.000
HNI	0.038	0.030	0.039	0.002	0	0.000	0.000
TRK	0.129	0.070	0.112	0.031	0.049	0	0.000
SDK	0.047	0.041	0.073	0.032	0.020	0.075	0

**Table 4:** Analysis of molecular variances (AMOVA) of microsatellites between the wild group and cultured group of the orange-spotted grouper in China.

Source of variation	Sum of squares	Variance components	Percentage variation	P value
Among groups	18.448	0.047	1.381	0.014
Among populations with groups	43.486	0.102	2.987	0.030
Among individuals within populations	623.593	−0.027	−0.801	−0.008
Within individuals	657.000	3.284	96.433	0.036
Total	1342.527	3.406	100.000	
